# A Systematic Review of Heterotopic Ossification Following Shoulder Arthroplasty: Is There a Clinical Value?

**DOI:** 10.7759/cureus.47374

**Published:** 2023-10-20

**Authors:** Christos G Dragonas, George Mamarelis, Shahan Shahid, Dimitrios Tsekes

**Affiliations:** 1 Trauma and Orthopaedics, The Princess Alexandra Hospital NHS Trust, Harlow, GBR; 2 Trauma and Orthopaedics, University of Calgary, Calgary, CAN; 3 Trauma and Orthopaedics, King’s College Hospital NHS Foundation Trust, London, GBR; 4 Trauma and Orthopaedics, Queen’s Hospital, London, GBR

**Keywords:** shoulder arthroplasty/replacement, shoulder, constant-murley score, shoulder hemiarthroplasty, range of motion (rom), osteophytes, heterotopic ossification

## Abstract

This systematic review aims to assess the contemporary literature on the incidence rate, functional scores, and clinical outcomes of heterotopic ossification (HO) following shoulder arthroplasty. We conducted a thorough literature search on EMBASE, MEDLINE, and Ortho Search to identify studies that directly compared patients with and without HO following hemiarthroplasty, anatomic total shoulder arthroplasty (aTSA), or reverse total shoulder arthroplasty (rTSA). We included studies with a minimum follow-up period of one year published from January 2000 to March 2023. We excluded case reports, editorials, and reviews. We performed the systematic review in accordance with Preferred Reporting Items for Systematic Reviews and Meta-Analyses guidelines. Of the 297 studies initially identified, seven met our inclusion criteria. These studies evaluated a total of 1,134 arthroplasties (212 hemiarthroplasties, 376 aTSAs, and 546 rTSAs). The mean follow-up period was 30.52 (12-120) months and the mean age was 68.69 (20-92). The overall incidence rate of HO was 26.8% (304/1,134). Male gender was associated with a higher incidence rate of HO in three studies. No statistically significant difference was noted in symptoms at follow-up or in Constant-Murley score at two years of follow-up (HO vs. non-HO: 70 vs. 75, p = 0.081). Only one study reported a significant difference in the post-arthroplasty range of motion, specifically in forward elevation (121° vs. 133°, p = 0.0087) and external rotation (19° vs. 25°, p-value = 0.0266). We conclude that HO after shoulder arthroplasty does not display a significant impact on postoperative symptoms or functional outcomes in the majority of patients. To fully comprehend its effect, further research and consensus among experts is necessary.

## Introduction and background

Heterotopic ossification (HO) is widely considered to be a common postoperative complication following shoulder arthroplasty surgery [1-3]. It is characterized by the formation of atypical bone outside the normal skeletal system, including skeletal muscles and fibrous connective tissues adjacent to joints [4,5].

Although this clinical entity can be encountered with different names in the current literature, such as paraosteoarthropathy, ectopic ossifications, or heterotopic bone formation/bony spurs, it is now most appropriately described with the term HO [6].

The incidence of HO following hemiarthroplasties, anatomic total shoulder arthroplasties (aTSAs), or reverse total shoulder arthroplasties (rTSAs) varies significantly, ranging between 6.6% and 54%, according to previous studies [5,7-10].

Multiple classification systems have been described for HO [7,11,12]. The most well-known and widely used system was initially described by Brooker et al. for hip HO [12], which has been further modified and modernized [4], as well as the one described by Kjaersgard-Andersen et al. [7].

Shoulder HO following shoulder arthroplasty has been extensively referenced in the literature, yet its clinical significance remains unclear. Some studies have suggested that the presence of HO, particularly low-grade HO, does not have an impact on the clinical outcome [7,13]. However, it has also been observed that certain cases of HO can potentially affect functional outcomes and may be mistaken for other radiographic changes, such as scapular notching [14]. This raises the question of whether HO has any clinical significance on functional and radiological outcomes.

In this systematic review, we conducted a thorough analysis of existing literature on the demographics, incidence, and clinical outcomes of HO of the proximal humerus/shoulder joint after shoulder arthroplasty (hemiarthroplasty or TSA). Our objective was to determine the potential clinical impact of HO.

## Review

Methodology

This study was designed and executed in accordance with Preferred Reporting Items for Systematic Reviews and Meta-Analyses (PRISMA) guidelines and protocols. The eligibility criteria were established before commencing the literature search.

Study Selection

A comprehensive literature search was performed on three databases (Embase, MEDLINE, Ortho Search) for studies that were published from January 2000 to May 2023 that compared clinical outcomes of patients with and without HO following shoulder replacement surgery (hemiarthroplasty, aTSA, or rTSA).

The following search terms were utilized: (“heterotopic ossification” AND “Shoulder”). Our analysis included studies that had English as their primary language or were published in the English language.

Two independent reviewers (CD, GM) assessed the identified studies’ titles, abstracts, and full texts following duplicate removal. Any discrepancies between the two reviewers were discussed and resolved by a third reviewer (GM).

The following inclusion criteria were used to screen the initial records: (1) Comparative studies that performed a direct comparison of patients with and without HO; (2) patients who underwent hemiarthroplasty, aTSA, or rTSA; and (3) follow-up greater than one year. We excluded studies that (1) included patients with less than one year of follow-up; (2) included shoulder replacement performed secondary to malignancy; (3) studies that lacked a comparison group; and (4) studies published in languages other than English.

We only included randomized controlled trials, cohort studies, and case-control studies. Biomechanical studies, technical notes, letters to the editor, expert opinions, review articles, meta-analyses, conference abstracts, and case reports were excluded.

Quality Assessment of Included Studies

The Methodological Index for Non-randomized Studies (MINORS) criteria were utilized to assess the quality of the included studies [15]. Each of the 12 items in the MINORS criteria is scored between 0 and 2, with maximum scores of 12 and 24 for non-comparative and comparative studies, respectively.

Results

From the initial literature search, 297 relevant studies were identified. Following the removal of 53 duplicate abstracts and the further removal of 42 abstracts that were published before 2000, 202 studies were screened. Of these, 163 were removed following abstract and title assessment. Consequently, a full-text review was conducted on 39 studies.

**Figure 1:**
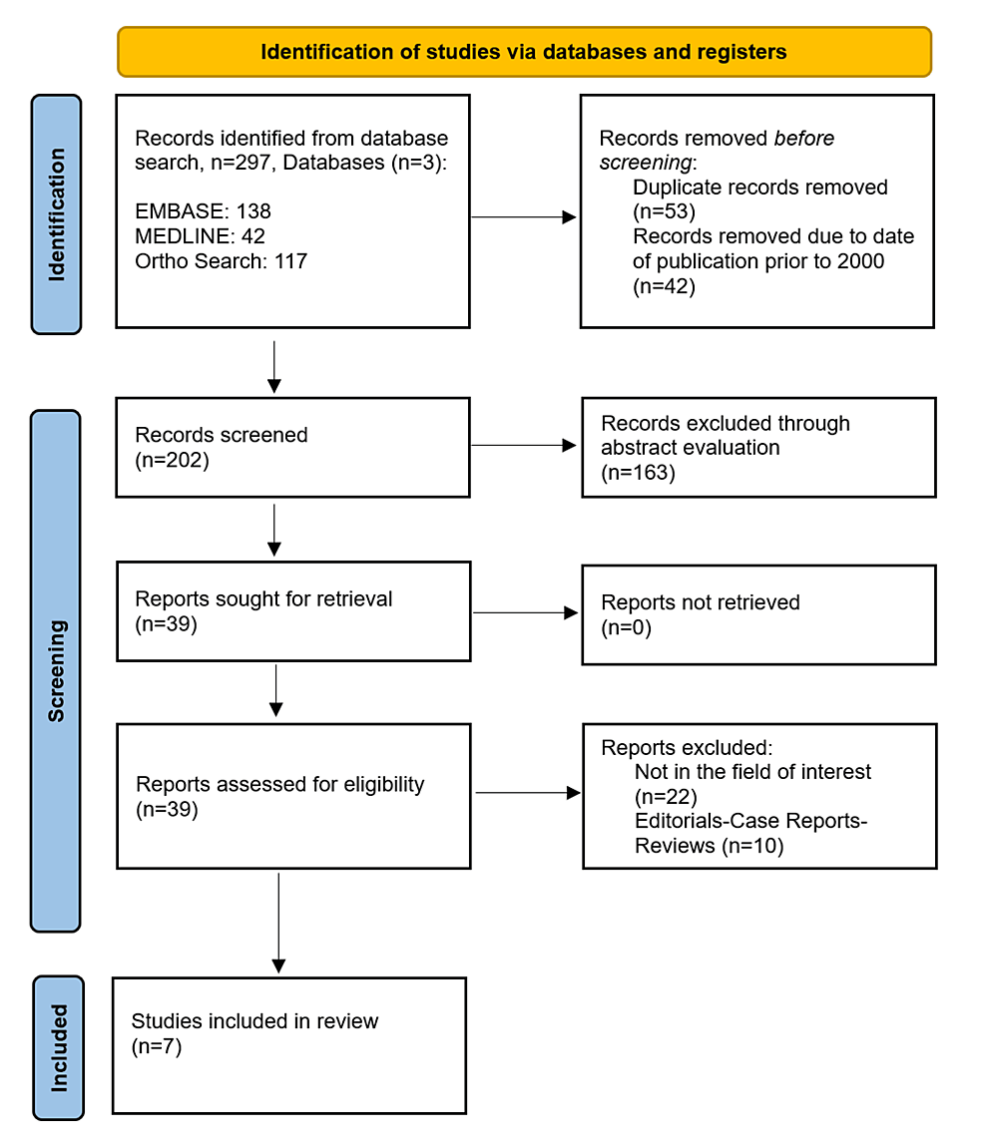
Preferred Reporting Items for Systematic Reviews and Meta-Analyses flowchart.

Five studies concerned case reports, four were letters to the editor/editorials, one concerned a short review, and 22 did not meet our inclusion criteria. This review includes seven studies that met all the aforementioned inclusion criteria [1,5,8,9,11,16,17] with a total of 1,133 patients (1,134 shoulder arthroplasties). The study selection process is outlined in the flowchart in Figure 1.

The mean follow-up duration was 30.52 (12-120) months, while the mean age of the patients was 68.69 (20-92) years. The full details of the included studies are summarized in Table 1.

**Table 1:** Studies included in the review. HO: heterotopic ossification; aTSA: anatomic total shoulder arthroplasty; rTSA: reverse total shoulder arthroplasty; ROM, range of motion; HO: heterotopic ossification; ASES: American Shoulder and Elbow Surgeons; CS: Constant-Murley score

Authors	Year	Total number of patients included in study	Mean follow-up duration (months)	Type of procedure performed	Number of patients with HO	Incidence rate	Mean age (years)	Clinical outcome scores
Sperling et al. [8]	2000	58	56 (24–120)	aTSA	14	24%	63 (28–80)	Neer result rating system, ROM, Symptoms at follow-up
Mighell et al. [17]	2003	71 (72 shoulders)	36 (12–89)	Hemiarthroplasty	18	25%	66 (39–89)	ASES score
Boehm et al. [5]	2004	126	26	Hemiarthroplasty (58); aTSA (68)	19	15%	62 (20–90)	Neer result rating system
Gronhagen et al. [9]	2007	82	52	Hemiarthroplasty	25	54%	72 (42–91)	ROM, CS
Verhofste et al. [1]	2016	132	36 (12–84)	rTSA	39	45%	69 (49–89)	CS
Kevin Ko et al. [11]	2016	164	24.26 (12–72)	rTSA	101	61.6%	71.1 (43–92)	ROM
Olsen et al. [16]	2020	500	25	aTSA (250); rTSA (250)	88	17.6%	70 (42–89)	ROM, symptoms at follow-up
Total	-	1,133 (1,134 shoulders)	30.52 (12–89)	Hemiarthroplasty (212); aTSA (376); rTSA (546)	304	26.8%	68.69 (20–92)	-

Methodological Quality Assessment

The mean MINORS score for the methodological quality assessment was 10/16 (range = 9-12). All studies had a retrospective design and subsequently lost marks for not having a prospective collection of data or prospective calculation of study size. Although all but one study provided methods of reliably and fairly marking their endpoint results, only one study ensured that interpreters were blinded. Two studies lost 1 point each for having an unsatisfactory number of patients lost to follow-up, and two studies lost a point each for not outlining a clear exclusion criterion for their patient groups (Table 2).

**Table 2:** Methodological Items for Non-randomized Studies (MINORS) score. The items are scored 0 (not reported), 1 (reported but inadequate), or 2 (reported and adequate). The global ideal score is 16 for non-comparative studies and 24 for comparative studies.

Methodological Items for Non-randomized Studies (MINORS)	Sperling et al., 2000 [8]	Mighell et al., 2003 [17]	Boehm et al., 2004 [5]	Grönhagen et al., 2007 [9]	Verhofste et al., 2016 [1]	Kevin Ko et al., 2016 [11]	Olsen et al., 2020 [16]
1. A clearly stated aim	2	2	2	2	2	2	2
2. Inclusion of consecutive patients	2	2	1	1	2	2	2
3. Prospective collection of data	0	0	0	0	0	0	0
4. Endpoints appropriate to the aim of the study	2	2	2	2	2	2	2
5. Unbiased assessment of the study endpoint	1	1	0	1	1	1	2
6. Follow-up period appropriate to the aim of the study	2	2	2	2	2	2	2
7. Loss to follow-up <5%	2	1	2	1	2	2	2
8. Prospective calculation of the study size	0	0	0	0	0	0	0
9. An adequate control group	N/A	N/A	N/A	N/A	N/A	N/A	N/A
10. Contemporary groups	N/A	N/A	N/A	N/A	N/A	N/A	N/A
11. Baseline equivalence of groups	N/A	N/A	N/A	N/A	N/A	N/A	N/A
12. Adequate statistical analysis	N/A	N/A	N/A	N/A	N/A	N/A	N/A
Total (Out of 24)	11	10	9	9	11	11	12

Data Analysis

The first relevant study that met the eligibility criteria and was included in our review was conducted in 2000 by Sperling et al. [8]. It included 58 patients who underwent TSA with an average age of 63 years (28-80 years) and a mean follow-up of 4.7 years. The research team utilized a classification system previously reported by Kjaersgaard-Andersen et al. [7], while they also graded pain and patient satisfaction according to a modified Neer result rating system [18,19]. Their results showed that 14 out of 58 patients (Grade I: 12 patients, Grade II: 2 patients) developed HO (incidence rate of 24%). No identifiable preoperative patient characteristics were associated with the development of HO. There was no statistically significant difference in the range of motion (ROM) between patients with and without HO (abduction: 135 vs. 140, p = 0.693; external rotation: 56 vs. 57, p = 0.687; internal rotation: second lumbar vertebrae vs. 12th thoracic vertebrae, p = 0.067). No significant difference was noted in postoperative pain (p = 0.108) or Neer result rating (p = 0.720) between the two groups.

Two studies in our review reported results solely focused on HO following hemiarthroplasty. The first one was conducted in 2007 by Mighell et al. [17] and included outcomes of 72 shoulders (71 patients) at a minimum follow-up of two years. They reported 18 cases of HO in their population (25%), and further classified them according to Brooker classification for hip HO [12] as Grade I for eight patients, Grade II for four patients, Grade III for four patients, and Grade IV for one patient. In all but one case, the anatomic location of the heterotopic bone formation was inferior to the head segment and medial to the humeral shaft, while in one case, it was observed at the undersurface of the deltoid. Although the team did not report any specific results or functional outcomes, they noted that one patient with Grade IV HO developed complete ankylosis of the joint and had to undergo revision surgery six months after the initial procedure. In 2007, in a similar study, Gronhagen et al. [9] included 46 patients (nine males, 37 females) who underwent primary hemiarthroplasty for a proximal humerus fracture with a mean age of 72 years (range = 42-91 years). They observed ectopic bone formation in 25 patients (incidence rate of 54%); however, all of them except one were found to have low-grade HO, without any significant correlation to ROM or difference in postoperative Constant-Murley score between the two groups.

In another study conducted by Boehm et al. [5] at Nottingham, UK, the incidence of HO following primary hemiarthroplasty or aTSA was assessed. In total, 126 shoulder joint replacements (hemiarthroplasties: 58, TSA: 68) were included with a mean age of 62 (20-90) years and an average follow-up of 26 months. This study group also utilized the previously mentioned classification system by Kjaersgaard-Andersen et al. [7]. They reported an incidence rate of 15% (19/126 shoulders). There was no significant difference between male (6/41, 14.6%) and female patients (13/85, 15.3%), while HO occurrence was not associated with the type of operation (hemiarthroplasty vs. TSA: 15.0% vs. 14.7%, p = 0.98). The study group noted that the only factor that was associated with a higher incidence of HO was the presence of cuff tear arthropathy (36.4%); however, due to the small number of patients with this diagnosis, the results were statistically insignificant (p = 0.39).

In 2016, Verhofste et al. [1] conducted a single-center retrospective study focusing on the incidence and clinical consequences of HO in 132 patients (36 males, 96 females) following rTSA with a mean follow-up of 36 months (range = 12 to 84 months). The study included 39 patients with HO (incidence rate of 29.5%) and a mean age of 69 years (range = 49-89 years), which developed over a mean period of 8.3 months (range = 3 to 21 months) and then stabilized without any further change. A modified Brooker classification system of HO of the hip was used for grading [4]. Male gender was the only reported factor associated with a higher incidence rate of HO (female vs. male, 22/96 vs. 17/36, p = 0.01). At two years of follow-up, the difference in CS was no longer statistically significant (p = 0.088). No significant difference was noted in the two-year follow-up in ROM (p = 0.97), power (p = 0.29), and pain (p = 0.29) either between patients with Grade II HO and those with no ossification present radiographically [1].

Additionally, in another retrospective study conducted by Ko et al. [11], a radiographic chart review was performed on 164 patients (mean age = 71.1 ± 9 years) who underwent rTSA with a Grammont-style prosthesis. The study aimed to evaluate the incidence, predisposing factors, and clinical relevance of HO appearing in the long head of the triceps. The research team also proposed a new grading system to measure the size of any HO within the region, classifying it into two types (type I: small, type II: large) and four subtypes (A: impinging, B: free-floating, C: concurrent notching, D: ankylosis) [11]. The overall rate of HO in the long head of the triceps tendon was 61.6%, with 31.7% being type I and 29.9% being type II. The rate of impinging HO was 23.2%, while the rate of notching was 14.6%. Additionally, the rate of free-floating osteophytes was 14.0%, and the rate of ankylosis was 3.0%. The team found that the male gender had a higher incidence of HO formation (male vs. female 74.0% vs. 56.1%; p = 0.0304), body mass index (>30 vs. <30, p = 0.3390), or cuff tear arthropathy (p = 0.4693). In terms of postoperative ROM, a statistically significant difference was observed in patients with HO compared to those without HO. Specifically, patients with HO had reduced forward elevation (121° vs. 133°, p = 0.0087) and external rotation (19° vs. 25°, p = 0.0266 in 140 patients). The size of the HO (type I vs. type II) and the presence of scapular notching did not affect postoperative ROM significantly.

Finally, in a recent study published by Olsen et al. in 2020 [16], 500 patients (266 males, 234 females) who underwent aTSA and rTSA were included with an average follow-up duration of 70.6 ± 8.2. The team included patients with HO that was observed anteriorly of the proximal humerus and near the expected attachment of the pectoralis major tendon. The study found that 88 patients developed HO, with an incidence rate of 17.6%. HO occurrence was significantly higher in males (25.4%) compared to females (8.5%) (p < 0.001), while aTSA was also associated with a higher incidence (25.2%) compared to rTSA (10.0%) (p < 0.001). However, there was no significant difference in reported symptoms (11.5% vs. 13.1%, p = 0.85) or postoperative ROM, including elevation (150 (145-160) vs. 160 (150-160), p = 0.42), external rotation (45 (40-50) vs. 45 (40-50), p = 0.52) and internal rotation (p = 0.057) between patients with or without HO.

Discussion

HO is a common radiological finding after arthroplasty, but its natural history and clinical relevance following shoulder arthroplasty have not been described adequately [10,20].

Age has been suggested as a risk factor for developing HO after any joint arthroplasty. In general, the development of HO following musculoskeletal trauma is more common in patients between the ages of 18-44, with less than 18% of patients over the age of 65. Possible explanations for this age-dependent tendency include a diminished inflammatory response with age, differences in the cells responsible for HO formation (mesenchymal stem cells), slowed bone remodeling and loss of bone quality, or variations in the severity of musculoskeletal trauma between older and younger patients [21]. On the other hand, it has been reported that patients over 65 who undergo joint arthroplasty may have an increased risk of developing HO [22]. Our review identified no correlation between age and HO following shoulder arthroplasty [1,5,8,16].

It has been suggested that a higher percentage of men tend to develop HO, indicating that gender could be a potential risk factor [1]. A possible reason could be the differences in muscle mass and hormonal signaling pathways that regulate osteogenesis [23]. However, our systematic review identified a controversy regarding whether gender plays a significant role in HO formation. Half of the authors reported no statistical difference between genders [5,8,9]. In contrast, the rest of the authors identified male gender as the sole factor linked to a high incidence of HO [1,11,16].

Another factor that can play a role in the increased risk of HO in shoulder arthroplasty is the choice of surgical approach (superolateral vs. deltopectoral) and the extent of operative release of soft tissues [3]. The degree of soft tissue release can be influenced by the type of shoulder replacement selected, whether it is aTSA or rTSA.

It is worth noting that rTSA inherently carries a higher risk of HO due to the extensive soft tissue release required [1]. However, Olsen et al. found that standard TSA has a higher risk of developing HO compared to rTSA, 25.2% vs. 10%, respectively [16]. This suggests that the correlation between HO and the choice of implant may be independent. Possibly attributed to variations in soft tissue releases, such as the complete or incomplete release of the long head of the triceps or partial release of the pectoralis major tendon [1,16]. Furthermore, Olsen et al. also noted that performing a tenotomy or subscapularis peeling carries a lower risk of HO compared to lesser tuberosity osteotomy [16]. In terms of surgical approach, Boehm et al. found no statistical difference between the deltopectoral and anterolateral approaches [5]. It is important to consider that they performed a deltopectoral approach for hemiarthroplasty and an anterolateral approach for aTSAs, each involving different soft tissue releases around the glenoid. In addition, Verhofste et al. [1] identified no statistical difference between the two approaches as well. In light of these findings, it becomes evident that surgeons must meticulously assess and plan the approach and soft tissue releases during shoulder arthroplasties to minimize the risk of HO for their patients.

An additional crucial factor to take into account when considering the formation of HO is the specific indication that leads to shoulder arthroplasty. Different indications, such as primary osteoarthritis (OA), rotator cuff arthropathy, rheumatoid arthritis (RA), or fracture, can have varying incidences on the formation of HO. Interestingly, a study by Boehm et al. found that cuff tear arthropathy had a higher occurrence of HO (up to 36.4%) compared to other primary diagnoses such as OA, fracture/dislocation, RA, and avascular necrosis [5]. Another study by Verhofste et al. reported a similar incidence (29.5%) of HO following shoulder arthroplasty for rotator cuff arthropathy [1]. Olsen et al. reported that degenerative arthritis has a higher risk of HO compared to cuff arthropathy with an incidence of 11% vs. 22.3%, respectively [16]. These findings underscore the importance of surgeons discussing these varying risks with patients during the informed consent process for shoulder arthroplasty.

There was no consensus in regard to the classification system of HO for shoulder arthroplasty. Few authors were using the Modified Brooker classification of the shoulder while others were using the Kharsgaard-Andersen classification system [1,7]. Additionally, Ko et al. have introduced a new system to classify the formation of traction osteophytes in the long head of the triceps tendon, providing further insight into this area [11]. The lack of consensus in the HO classification system for shoulder arthroplasty, with all variations, highlights the ongoing need for research and refinement.

There is ongoing debate surrounding the timing and method of how HO can impact the clinical outcomes of patients who undergo shoulder arthroplasty. Some studies suggest that HO formation following shoulder arthroplasty is a non-progressive situation that does not have a negative impact on the clinical outcomes [1,8,16]. However, we believe that the surgeon should always check for the traction osteophyte of the long head of the biceps, as this is associated with reduced postoperative motion [11].

## Conclusions

Our systematic review of HO following shoulder arthroplasty revealed several important factors that warrant consideration in clinical practice such as age, gender, surgical approach/soft tissue releases, and primary indications. Furthermore, the absence of a unified classification system underscores the ongoing need for research in this field. The debate regarding the clinical impact of HO formation continues. The traction osteophyte of the long head of the biceps may play an important role in optimizing postoperative outcomes. Further research and consensus among experts are needed to fully understand the influence of HO on shoulder arthroplasty outcomes.
